# Does Family Caregiver Self-Identification Matter? An Analysis of Survey Responses

**DOI:** 10.1093/geroni/igaf122.2354

**Published:** 2025-12-31

**Authors:** Pamela Nadash, Eileen Tell, Chang Pu Liang, Marc Cohen

**Affiliations:** University of Massachusetts Boston, Boston, Massachusetts, United States; ET Consulting, LLC, Bemont, Massachusetts, United States; University of Massachusetts Boston, Boston, Massachusetts, United States; University of Massachusetts Boston, Boston, Massachusetts, United States

## Abstract

One of the five goals identified as a priority by the National Strategy to Support Family Caregivers is Goal 1, which centers on raising awareness of family caregiving. While this goal concerns awareness-raising on all fronts, raising awareness among caregivers themselves may be the most important, given its presumed role in accessing supports. As part of an evaluation supporting the Administration for Community Living, 4,573 representative family caregivers across 10 states were surveyed on a range of issues in the Fall/early Winter of 2024. This study focuses on the extent to which they self-identified as caregivers, using both quantitative and qualitative data. It found that while only 66% of respondents initially identified themselves as caregivers, after interviewers defined the term, 74% did. An open-ended question asking respondents to define caregivers found that participants regularly mentioned paid caregivers, rarely mentioned end of life or dementia caregiving, and frequently referred to the daily nature of caregiving and types of tasks performed. Multivariate analyses found that self-identification was less likely amongst older and retired caregivers, men, lower income caregivers, and caregivers with less than a college education, although not among Hispanic caregivers. Controlling for factors including age and education, we found high-awareness caregivers were more likely to be confident about providing care and accessing services and supports than less aware caregivers were, and were more likely to agree that government and communities should play a role supporting caregivers. These findings underline the importance of caregiver self-identification in accessing services and in making change.

